# Supplementary material for: Higher predicted type 2 diabetes risk is associated with worse mental health and self-rated general health among adults without known diabetes in Germany – Results of the nationwide population-based study GEDA 2022
Source: PLoS One. 2025 Nov 7;20(11):e0336019. doi: 10.1371/journal.pone.0336019 (PMC12594385; doi:10.1371/journal.pone.0336019)
Supplement: S1 Table — Missing values: self-rated health (n = 1), self-rated mental health (n = 12), depressive symptoms (n = 80), anxiety symptoms (n = 60), educational level (n = 12), living alone (n = 8) and social support (n = 130). a Weighted prevalence ratios (PR) and 95% confidence intervals (95% CIs) were derived from separate Poisson regression models with self-rated health, self-rated mental health, depressive symptoms and anxiety symptoms as dependent variables. Model 4: adjusted additionally for educational level, region, living alone and social support. (DOCX) [file pone.0336019.s001.docx]

S1 Table. Prevalence ratio (95% CI) for the association of T2D risk with self-rated health, self-rated mental health, depressive symptoms, and anxiety symptoms among adults without diabetes, multiple imputation (n=4,909)

|  | **Categories of T2D risk** | | | |
| --- | --- | --- | --- | --- |
|  | **Very low risk (<2%)** | **Low risk (2% to <5%)** | **Elevated risk (5% to <10%)** | **High risk (≥10%)** |
|  | PR^a^ (95% CI) | PR^a^ (95% CI) | PR^a^ (95% CI) | PR^a^ (95% CI) |
| **Very good/good self-rated health (SRH)** |  |  |  |  |
| Model 4 | 1 (reference) | 0.81 (0.73-0.89) | 0.83 (0.73-0.94) | 0.65 (0.56-0.74) |
| **Excellent/very good self-rated mental health (SRMH)** |  |  |  |  |
| Model 4 | 1 (reference) | 0.84 (0.72-0.98) | 0.76 (0.62-0.92) | 0.67 (0.53-0.84) |
| **Depressive symptoms** |  |  |  |  |
| Model 4 | 1 (reference) | 2.01 (1.40-2.90) | 2.55 (1.71-3.81) | 2.55 (1.76-3.69) |
| **Anxiety symptoms** |  |  |  |  |
| Model 4 | 1 (reference) | 1.52 (0.91-2.54) | 2.12 (1.19-3.76) | 2.54 (1.58-4.09) |

Missing values: self-rated health (n=1), self-rated mental health (n=12), depressive symptoms (n=80), anxiety symptoms (n=60), educational level (n=12), living alone (n=8) and social support (n=130)

^a^ Weighted prevalence ratios (PR) and 95% confidence intervals (95% CIs) were derived from separate Poisson regression models with self-rated health, self-rated mental health, depressive symptoms and anxiety symptoms as dependent variables. Model 4: adjusted additionally for educational level, region, living alone and social support
