# Supplementary material for: Higher predicted type 2 diabetes risk is associated with worse mental health and self-rated general health among adults without known diabetes in Germany – Results of the nationwide population-based study GEDA 2022
Source: PLoS One. 2025 Nov 7;20(11):e0336019. doi: 10.1371/journal.pone.0336019 (PMC12594385; doi:10.1371/journal.pone.0336019)
Supplement: S2 Table — Missing values: self-rated health (n = 1), self-rated mental health (n = 12), depressive symptoms (n = 80), anxiety symptoms (n = 60). (DOCX) [file pone.0336019.s002.docx]

**S2 Table. Prevalence (95% CI) of self-rated health, self-rated mental health, depressive symptoms and anxiety symptoms by age among adults without diabetes (n=4,909)**

|  | Age group | | |  |
| --- | --- | --- | --- | --- |
|  | 18-44 years | 45-64 years | ≥65 years |  |
|  | % (95% CI) | % (95% CI) | % (95% CI) | p-value |
| Very good/good self-rated health (SRH) | 83.5 (80.0-86.5) | 68.6 (65.0-72.0) | 56.8 (53.3-60.3) | <0.001 |
| Excellent/very good self-rated mental health (SRMH) | 44.2 (40.1-48.3) | 41.1 (37.7-44.5) | 32.8 (29.6-36.2) | <0.001 |
| Depressive symptoms | 16.3 (13.3-19.8) | 14.6 (11.9-17.8) | 10.7 (8.6-13.2) | 0.036 |
| Anxiety symptoms | 12.9 (10.2-16.2) | 11.5 (9.1-14.4) | 6.8 (5.2-8.9) | 0.008 |

Missing values: self-rated health (n=1), self-rated mental health (n=12), depressive symptoms (n=80), anxiety symptoms (n=60)
