# Supplementary material for: Higher predicted type 2 diabetes risk is associated with worse mental health and self-rated general health among adults without known diabetes in Germany – Results of the nationwide population-based study GEDA 2022
Source: PLoS One. 2025 Nov 7;20(11):e0336019. doi: 10.1371/journal.pone.0336019 (PMC12594385; doi:10.1371/journal.pone.0336019)
Supplement: S3 Table — Missing values: self-rated health (n = 1), self-rated mental health (n = 12), depressive symptoms (n = 80), anxiety symptoms (n = 60), educational level (n = 12), living alone (n = 8) and social support (n = 130). 1 Weighted prevalence ratios (PR) and 95% confidence intervals (95% CIs) were derived from separate Poisson regression models with self-rated health, self-rated mental health, depressive symptoms and anxiety symptoms as dependent variables. Model 1: unadjusted; Model 2: adjusted for sex; Model 3: adjusted additionally for age; Model 4: adjusted additionally for educational level, region, living alone and social support. (DOCX) [file pone.0336019.s003.docx]

**S3 Table. Prevalence ratio (95% CI) for the association of continuous T2D risk with self-rated health, self-rated mental health, depressive symptoms, and anxiety symptoms among adults without diabetes (n=4,909)**

|  | T2D risk score  (logarithmically transformed) |
| --- | --- |
|  | PR^1^ (95% CI) |
| **Very good/good self-rated health (SRH)** |  |
| Model 1 | 0.89 (0.88-0.90) |
| Model 2 | 0.89 (0.87-0.90) |
| Model 3 | 0.87 (0.85-0.90) |
| Model 4 | 0.89 (0.87-0.92) |
| **Excellent/very good self-rated mental health (SRMH)** |  |
| Model 1 | 0.89 (0.86-0.91) |
| Model 2 | 0.88 (0.85-0.90) |
| Model 3 | 0.81 (0.77-0.85) |
| Model 4 | 0.84 (0.80-0.89) |
| **Depressive symptoms** |  |
| Model 1 | 1.08 (1.01-1.16) |
| Model 2 | 1.09 (1.01-1.17) |
| Model 3 | 1.35 (1.23-1.48) |
| Model 4 | 1.23 (1.11-1.37) |
| **Anxiety symptoms** |  |
| Model 1 | 1.08 (1.00-1.18) |
| Model 2 | 1.10 (1.01-1.20) |
| Model 3 | 1.44 (1.29-1.61) |
| Model 4 | 1.34 (1.19-1.50) |

Missing values: self-rated health (n=1), self-rated mental health (n=12), depressive symptoms (n=80), anxiety symptoms (n=60), educational level (n=12), living alone (n=8) and social support (n=130)

^1^ Weighted prevalence ratios (PR) and 95% confidence intervals (95% CIs) were derived from separate Poisson regression models with self-rated health, self-rated mental health, depressive symptoms and anxiety symptoms as dependent variables. Model 1: unadjusted; Model 2: adjusted for sex; Model 3: adjusted additionally for age; Model 4: adjusted additionally for educational level, region, living alone and social support
