# Supplementary material for: Higher predicted type 2 diabetes risk is associated with worse mental health and self-rated general health among adults without known diabetes in Germany – Results of the nationwide population-based study GEDA 2022
Source: PLoS One. 2025 Nov 7;20(11):e0336019. doi: 10.1371/journal.pone.0336019 (PMC12594385; doi:10.1371/journal.pone.0336019)
Supplement: S4 Table — Missing values: self-rated health (n = 1), self-rated mental health (n = 12), depressive symptoms (n = 80), anxiety symptoms (n = 60), educational level (n = 12), living alone (n = 8) and social support (n = 130). 1 p-values were derived from Poisson regression model with self-rated health, self-rated mental health, depressive symptoms and anxiety symptoms as dependent variables. Adjusted for educational level, region, living alone and social support. (DOCX) [file pone.0336019.s004.docx]

**S4 Table. Interaction of continuous T2D risk score with sex and age for self-rated health, self-rated mental health, depressive symptoms, anxiety symptoms and among people without diabetes (Prevalence ratio and 95% CI) (n=4,909)**

|  | **Interaction of T2D risk score (logarithmically transformed) with sex** | **Interaction of T2D risk score (logarithmically transformed) with age** |
| --- | --- | --- |
|  | **p-value**^1^ | **p-value**^1^ |
| **Very good/good self-rated health (SRH)** | 0.515 | 0.018 |
| **Excellent/very good self-rated mental health (SRMH)** | 0.831 | 0.270 |
| **Depressive symptoms** | 0.454 | 0.396 |
| **Anxiety symptoms** | 0.337 | 0.742 |

Missing values: self-rated health (n=1), self-rated mental health (n=12), depressive symptoms (n=80), anxiety symptoms (n=60), educational level (n=12), living alone (n=8) and social support (n=130)

^1^ p-values were derived from Poisson regression model with self-rated health, self-rated mental health, depressive symptoms and anxiety symptoms as dependent variables. Adjusted for educational level, region, living alone and social support
