# Supplementary material for: Higher predicted type 2 diabetes risk is associated with worse mental health and self-rated general health among adults without known diabetes in Germany – Results of the nationwide population-based study GEDA 2022
Source: PLoS One. 2025 Nov 7;20(11):e0336019. doi: 10.1371/journal.pone.0336019 (PMC12594385; doi:10.1371/journal.pone.0336019)
Supplement: S5 Table — *p < 0.05. Missing values: self-rated health (n = 1), self-rated mental health (n = 12), depressive symptoms (n = 80), anxiety symptoms (n = 60), educational level (n = 12), living alone (n = 8) and social support (n = 130). (DOCX) [file pone.0336019.s005.docx]

**S5 Table. Pairwise correlations between age, T2D risk and self-rated health, self-rated mental health, depressive symptoms, anxiety symptoms and among people without diabetes (n=4,909)**

|  | **T2D risk** | **Very good/good self-rated health (SRH)** | **Excellent/very good self-rated mental health (SRMH)** | **Depressive symptoms** | **Anxiety symptoms** | | **Age** |
| --- | --- | --- | --- | --- | --- | --- | --- |
| **T2D risk** | 1.000 |  |  |  |  |  | |
| **Very good/good self-rated health (SRH)** | -0.292* | 1.000 |  |  |  |  | |
| **Excellent/very good self-rated mental health (SRMH)** | -0.137* | 0.317* | 1.000 |  |  |  | |
| **Depressive symptoms** | 0.068* | -0.280* | -0.270* | 1.000 |  |  | |
| **Anxiety symptoms** | 0.047* | -0.258* | -0.256* | 0.482* | 1.000 |  | |
| **Age** | 0.619* | -0.256* | -0.095* | -0.055* | -0.059* | 1.000 | |

*p<0.05

Missing values: self-rated health (n=1), self-rated mental health (n=12), depressive symptoms (n=80), anxiety symptoms (n=60), educational level (n=12),
living alone (n=8) and social support (n=130)
