# Supplementary material for: Higher predicted type 2 diabetes risk is associated with worse mental health and self-rated general health among adults without known diabetes in Germany – Results of the nationwide population-based study GEDA 2022
Source: PLoS One. 2025 Nov 7;20(11):e0336019. doi: 10.1371/journal.pone.0336019 (PMC12594385; doi:10.1371/journal.pone.0336019)
Supplement: Attachment — Submitted filename: Response to Editor and Reviewers.docx [file pone.0336019.s007.docx]

## Journal Requirements:

When submitting your revision, we need you to address these additional requirements.

1. Please ensure that your manuscript meets PLOS ONE's style requirements, including those for file naming. The PLOS ONE style templates can be found at

<https://journals.plos.org/plosone/s/file?id=wjVg/PLOSOne_formatting_sample_main_body.pdf> and

<https://journals.plos.org/plosone/s/file?id=ba62/PLOSOne_formatting_sample_title_authors_affiliations.pdf>

**Response:** We have revised the manuscript to ensure that it fully complies with the PLOS ONE style requirements, including formatting and file naming conventions.

2. In the ethics statement in the Methods, you have specified that verbal consent was obtained. Please provide

additional details regarding how this consent was documented and witnessed, and state whether this was approved by the IRB

**Response:** As stated and now extended in the Methods section (manuscript with tracked changes lines 106-111) and in the Ethics Statement, the study was conducted in adherence to the principles of the Helsinki Declaration [35] and the guidelines provided by the Federal Commissioner for Data Protection and Freedom of Information in Germany. It was approved by the Charité, Universitätsmedizin Berlin local ethics committee (No. EA2/201/21). All participants in the study gave their verbal consent to the storage of their data and its anonymised transfer to the RKI (‘informed consent’). Consent was based on the free decision of the person called, who was informed of the voluntary nature of participation and the anonymised processing of the data. Verbal consent was obtained after the interviewer had read aloud the data protection consent form; the respondent’s agreement was documented electronically by recording the value “1” in the survey database, and the interviewer acted as the witness of the consent procedure. This procedure, including its documentation, was approved by the Charité ethics committee (No. EA2/201/21), which served as the competent Institutional Review Board (IRB). This complies with Article 7(1) General Data Protection Regulation (“Datenschutz-Grundverordnung, DSGVO”) regarding the verifiability of consent by the responsible site. If the respondent did not consent, the interview was not conducted. The use of verbal consent in accordance with the procedure described is an established standard practice in nationwide telephone health surveys in Germany.

3. Thank you for stating the following financial disclosure:

“This work was supported by a grant from the German Center for Diabetes Research (DZD) funded by the German Federal Ministry of Education and Research (grant number: HMGU2022Z5) and grants from the German Federal Ministry of Health within the framework of the National Diabetes Surveillance project at the Robert Koch Institute (grant numbers: 2522DIA700 and 2523DIA002). The survey GEDA 2022 was funded by the Robert Koch Institute and the Federal Ministry of Health in Germany.”

Please state what role the funders took in the study. If the funders had no role, please state: "The funders had no role in study design, data collection and analysis, decision to publish, or preparation of the manuscript."

If this statement is not correct you must amend it as needed.

Please include this amended Role of Funder statement in your cover letter; we will change the online submission form on your behalf.

**Response:** We included the Statement in the Cover Letter as well as in the Statement of “Funding sources” at the end of the manuscript (manuscript with tracked changes lines 475-476).

4. We noted in your submission details that a portion of your manuscript may have been presented or published elsewhere. “Parts of the results have previously been presented in abstract form at two scientific conferences (a German-language abstract for a national joint public health conference in Dresden [1], and an English-language poster at the 17th European Public Health Conference [2]). These prior formats do not constitute formal publication, and the full manuscript is original and has not been published or submitted elsewhere.

1. Neuperdt L, Du Y, Nübel J, Hapke U, Walther L, Mensink GBM, et al. Assoziation des Typ-2-Diabetes-Risikos mit selbsteingeschätzter allgemeiner und psychischer Gesundheit bei Erwachsenen in Deutschland – Ergebnisse der Studie GEDA 2022 Gesundheit – gemeinsam Kooperationstagung der Deutschen Gesellschaft für Medizinische Informatik, Biometrie und Epidemiologie (GMDS), Deutschen Gesellschaft für Sozialmedizin und Prävention (DGSMP), Deutschen Gesellschaft für Epidemiologie (DGEpi), Deutschen Gesellschaft für Medizinische Soziologie (DGMS) und der Deutschen Gesellschaft für Public Health (DGPH); Dresden, Germany German Medical Science GMS Publishing House; 2024. doi: 10.3205/24gmds625

2. Neuperdt L, Du Y, Nübel J, Hapke U, Walther L, Mensink GBM, et al. Type 2 diabetes risk is associated with poorer general health and mental health in women and men. 17th European Public Health Conference 2024; Lisbon, Portugal: European Journal of Public Health; 2024. doi: 10.1093/eurpub/ckae144.1291”

Please clarify whether this [conference proceeding or publication] was peer-reviewed and formally published. If this work was previously peer-reviewed and published, in the cover letter please provide the reason that this work does not constitute dual publication and should be included in the current manuscript.

**Response:** Thank you for the opportunity to clarify. Parts of the results were disseminated as published conference abstracts (Neuperdt et al., GMDS/DGPH 2024; Neuperdt et al., EPHC 2024). These outputs represent only brief conference abstracts/poster presentations with preliminary results and were not peer-reviewed, and therefore do not constitute prior publication or dual submission. We disclosed these abstracts in the original submission for transparency and have added the above statement to the Cover Letter to make this explicit.

4. You have indicated that data is available from [fdz@rki.de]. Please can we ask you to provide us with a general contact email address for the data requests, so readers can request access in perpetuity. If a general email is not available please provide a link to a website where readers can obtain access to data.

**Response:** The general, permanent contact for data requests is the RKI Research Data Center (FDZ) at [fdz@rki.de](mailto:fdz@rki.de). Readers can also find access information and request procedures on the FDZ website: <https://www.rki.de/EN/News/Publications/Research-data/Scientific-Use-Files/scientific-use-files-node.html>

We added a sentence in the manuscript (manuscript with tracked changes lines 463-465).

5. Please include captions for your Supporting Information files at the end of your manuscript, and update any in-text citations to match accordingly. Please see our Supporting Information guidelines for more information: <http://journals.plos.org/plosone/s/supporting-information>.

**Response:**

We have added captions for all Supporting Information files at the end of the manuscript and updated the in-text citations accordingly, in line with the PLOS ONE Supporting Information guidelines.

6. If the reviewer comments include a recommendation to cite specific previously published works, please review and evaluate these publications to determine whether they are relevant and should be cited. There is no requirement to cite these works unless the editor has indicated otherwise.

**Response:** The reviewers did not recommend citing any specific previously published works.

## Additional Editor Comments:

Dear authors

The following points need explanation

1: Verbal consent is usually unacceptable, and simply will weaken the study. Add a brief sentence that using verbal consent will not weak the results

**Response:** We appreciate this comment. We have now added a sentence to the Methods section to clarify that verbal consent is an established standard procedure for nationwide telephone health surveys in Germany and is approved by the ethics committee (manuscript with tracked changes lines 111-113). Thus, it does not weaken the validity of the study results.

2: The data of waist circumference was generated from an equation using height, weight, sex, and age (ref. 37). Please add the marginal errors in using this equation

**Response:** Thank you for this helpful comment. Sex-specific means of calculated waist circumference were for men 95.9 cm (95% CI 95.0-96.8) and for women 84.7 cm (95% CI 83.9-85.5). The corresponding margin of error for the sample mean was ±0.90 cm in men and ±0.81 cm in women. We added this information in a sentence in the manuscript (manuscript with tracked changes lines 126-129).

## Reviewers' comments:

Reviewer's Responses to Questions

Comments to the Author

1. Is the manuscript technically sound, and do the data support the conclusions?

The manuscript must describe a technically sound piece of scientific research with data that supports the conclusions. Experiments must have been conducted rigorously, with appropriate controls, replication, and sample sizes. The conclusions must be drawn appropriately based on the data presented.

Reviewer #1: Yes

Reviewer #2: Partly

2. Has the statistical analysis been performed appropriately and rigorously?

Reviewer #1: Yes

Reviewer #2: No

3. Have the authors made all data underlying the findings in their manuscript fully available?

The PLOS Data policy requires authors to make all data underlying the findings described in their manuscript fully available without restriction, with rare exception (please refer to the Data Availability Statement in the manuscript PDF file). The data should be provided as part of the manuscript or its supporting information, or deposited to a public repository. For example, in addition to summary statistics, the data points behind means, medians and variance measures should be available. If there are restrictions on publicly sharing data—e.g. participant privacy or use of data from a third party—those must be specified.

Reviewer #1: No

Reviewer #2: Yes

4. Is the manuscript presented in an intelligible fashion and written in standard English?

PLOS ONE does not copyedit accepted manuscripts, so the language in submitted articles must be clear, correct, and unambiguous. Any typographical or grammatical errors should be corrected at revision, so please note any specific errors here.

Reviewer #1: Yes

Reviewer #2: No

5. Review Comments to the Author

Please use the space provided to explain your answers to the questions above. You may also include additional

comments for the author, including concerns about dual publication, research ethics, or publication ethics. (Please upload your review as an attachment if it exceeds 20,000 characters)

### Reviewer #1:

Higher predicted type 2 diabetes risk is associated with worse mental health and self-rated general

health among adults without known diabetes in Germany – Results of the nationwide population-based study GEDA 2022

Telephone interviews of around 5000 german adults. 5-year diabetes risk was determined with German Diabetes Risk Score and self rated health and mental health was examined depending on risk group. Depressive and anxiety symptoms were more common among those with high risk of diabetes. The study is soundly conducted, but may benefit from further highlighting how it adds to current knowledge.

Comments

**Major:**

1. Please add a characteristics table (as table 1) stratified by diabetes risk classification.

**Response:** Thank you for this helpful suggestion. We added a characteristics table as table 1 stratified by GDRS risk classification (manuscript with tracked changes line 237-238).

2. ”We hypothesized that individuals with a higher predicted 5-year T2D risk are less likely to have favourable SRH and SRMH”. This is not tested in Table 3. Instead you test excellent SRH and SRMH. Please harmonize hypothesis and test. Perhaps the outcomes tested in table 3 should be poos SRH and SRMH? Alternatively, that the hypothesis is revised to state that excellent SRH and SRMH are less common among those with high diabetes risk?

**Response:** We have revised the hypothesis wording to ensure consistency with the outcomes presented in Table 3. The hypothesis now states: “We hypothesized that individuals with a higher predicted 5-year T2D risk are less likely to have very good/good SRH and excellent/very good SRMH and are more likely to have depressive and anxiety symptoms.”

**Minor:**

1. It is stated in the introduction that diabetes is characterized by high glucose and caused by insulin resistance. An alternative statement would be that it is defined by high glucose and caused by a combination of life-style and genetic risk-factors. As you later discuss life-style it would be good to also highlight the importance of this factor in the introduction in a similar fashion as you now do for life-style and mental health.

**Response:** Thank you for your suggestion. We added the following sentence in the introduction: The pathogenesis of T2D involves a complex interplay of genetic predisposition, lifestyle and environmental factors (Zheng et al, 2018; Ley et al., 2016) (manuscript with tracked changes lines 54-55).

References:

Zheng Y, Ley SH, Hu FB. Global aetiology and epidemiology of type 2 diabetes mellitus and its complications. Nat Rev Endocrinol. 2018;14(2):88–98. Epub 20171208. doi: 10.1038/nrendo.2017.151.

Ley SH, Ardisson Korat AV, Sun Q, Tobias DK, Zhang C, Qi L, et al. Contribution of the Nurses' Health Studies to Uncovering Risk Factors for Type 2 Diabetes: Diet, Lifestyle, Biomarkers, and Genetics. Am J Public Health. 2016;106(9):1624–30. Epub 20160726. doi: 10.2105/ajph.2016.303314.

2. You state that you adress a research gap with this paper. Please present what is currently known about this overlap, and also present what current guidelines from EASD and ADA say about addressing mental health in diabetes.

**Response:** Regarding the research gap, we have clarified the evidence summary (manuscript with tracked changes lines 69-71) and now state explicitly that, while prior work has linked individual lifestyle-related factors to self-rated health and mental health and a few studies considered combinations of lifestyle factors and self-rated health as well as chronic stress, population-based analyses relating an overall risk profile of multiple health-related factors combined to self-rated health, self-rated mental health and to depressive and anxiety symptoms have not been reported.

Irrespective of this, we agree that guidelines are relevant to the broader topic of mental health in diabetes care. Therefore, we have also added a concise summary of current ADA / ADA–EASD recommendations on psychosocial care to the Discussion (see new paragraph: manuscript with tracked changes lines 407-412), and explicitly note that these guidelines primarily target persons with manifest diabetes; we then discuss the implication that evidence-based guidance for psychosocial screening or interventions in high-risk, but non-diabetic populations remains limited. References have been added (ADA psychosocial position statement; ADA Standards of Care; ADA–EASD consensus).

3. This statement in the discussion should be revised: ”epidemiological research on the association of T2D risk,

comprising multiple lifestyle-related and biological risk factors, with SRH is scarce.”. There is extensive research in this area, please rephrase or be more specific with objective statements of quantity.

**Response:** Thank you for this suggestion. We revised the text to improve precision and now state: While a link between SRH and manifest T2D is well established [5, 62, 63], epidemiological evidence directly examining validated T2D risk scores, comprising multiple lifestyle-related and biological risk factors, in relation to SRH in current nationwide population samples in Germany remains limited.

4. Please avoid the use of cardiometabolic health. This is not what you have been studying specifically. There are uncertainties in telephone interviews, predictive instruments and self reported health. Adding another layer of uncertainty by using a loosely defined term such as cardiometabolic health only increases the uncertainty of the study with no added benefit.

**Response:** We thank the reviewer for this valuable suggestion. We have avoided the term “cardiometabolic health” throughout the manuscript as far as possible and replaced it with more precise wording (either “physical health” or “T2D risk”, depending on the context).

### Reviewer #2:

**methods & statistics**

1) Clarify the regression specification (robust variance + survey design).

You analyze binary outcomes with Poisson regression to obtain prevalence ratios (PRs).

That’s fine if you’re using modified Poisson with a robust (sandwich) variance, ideally in a survey framework. Please state explicitly that you used robust standard errors and how the survey design was set (svyset in Stata: weights, PSUs, strata; variance estimator). At present it only says “the survey procedure” and “Poisson regression” without variance details, which matters for PR CIs and p-values [L159-L167][L171-L179]. See PLOS ONE’s statistical reporting guidance and standard references on modified Poisson.

**Response:** Thank you for this helpful comment. We have now stated the variance details explicitly. The survey design was specified in STATA as svyset [pweight = wQS_tmod]. wQS_tmod denoting the GEDA wave- and module-specific weight. Primary sampling units and strata were not part of the design of this telephone survey, so it was not necessary to account for clustering and stratification. Variances were estimated using Taylor linearization (Stata’s survey default). For bivariate associations we used the *Rao–Scott* chi-square test. For multivariable analyses, we estimated prevalence ratios (PRs) using modified Poisson regression with a log link and design-based (svy) variance estimation, i.e., Poisson models with robust/sandwich standard errors in a survey framework, as recommended in Stata manual for Poisson regression (StataCorp. Poisson regression (Stata-Manuals; Release 13). 2013 [cited 18.09.2025]. Available from: <https://www.stata.com/manuals13/rpoisson.pdf>.)

2) Justify the choice of covariates (risk-score components vs confounders).

The exposure (GDRS) already bundles age, smoking, diet, etc. When you additionally adjust for age (and sometimes variables causally related to lifestyle, e.g., social support), you risk overadjustment or conditioning on mediators. Please include a brief causal rationale (e.g., a DAG) for what you adjust for and why. PLOS encourages transparent confounder rationale in line with STROBE.

**Response:** Following this suggestion, we constructed a DAG to prespecify confounder control. To keep the manuscript concise, we provide the DAG in this response letter rather than as supplementary material; however, we are happy to move it to the Supplement if the reviewer and editor prefer. We treated the GDRS as a composite exposure and therefore did generally not adjust for its individual components to avoid over adjustment; instead, the primary adjustment focused on sociodemographic factors. Because age is part of the GDRS, but is associated with depressive symptoms and anxiety in an opposite direction compared to the GDRS, we implemented a stepwise model sequence with adding age not before Model 3 specifically to assess how age adjustment changes the GDRS-outcome association. The DAG was used solely to structure confounder control in associational analyses, and we refrain from causal claims given the cross-sectional design and potential bidirectionality. We added a sentence in the manuscript (manuscript with tracked changes lines 193-197).

**Fig. Directed acyclic graph (DAG) used to prespecify covariate adjustment.**
The DAG depicts hypothesised relations between the exposure (German Diabetes Risk Score, GDRS), outcomes (self-rated health [SRH], self-rated mental health [SRMH], depressive symptoms, anxiety symptoms), and covariates (sex, age, educational level, region, living alone, social support). Arrows indicate hypothesized directional dependencies used to identify confounding paths and define adjustment sets; they do not imply causation. Because T2D risk and mental health outcomes are bidirectionally related, the DAG encodes a predominant direction solely for adjustment in these cross-sectional analyses. Because age is part of the GDRS, we implemented a stepwise model sequence: after the unadjusted and sex-adjusted models, age was added in Model 3 specifically to assess how age adjustment changes the GDRS-outcome association. DAG created with DAGitty (dagitty.net).

3) Dealing with missing data.

Model 4 drops cases with missing education/living alone/social support (n=150; ~3%); please report the model specific analytic N directly in Table 3 and consider multiple imputation to limit bias if missingness isn’t completely at random [L64-L66][L243-L248].

**Response:** We thank the reviewer for this valuable suggestion. For transparency, we now report the model-specific analytic N directly in Table 3.

We also conducted multiple imputation for missing values in education, living alone, and social support (manuscript with tracked changes lines 200-203). The imputed models produced results highly consistent with the complete-case analyses. Because the differences between the complete-case and imputed models were negligible, we decided to retain the complete-case results as the main analyses, while providing the imputation results in the Supplementary Materials (S1 Table).

4) Bivariable vs adjusted inconsistencies (suppression by age).

You explain the anxiety finding as a suppression effect when adjusting for age—good. Consider adding a short

supplemental table with correlations between age, GDRS, and outcomes to make this concrete (readers unfamiliar with suppression will appreciate that) [L288-L294]. A one-sentence textbook-style note would help.

**Response:** We have added a brief supplemental correlation table (S5 Table) showing pairwise Pearson correlations between age, GDRS, and all outcomes. As shown in S5 Table, age is positively correlated with the exposure (GDRS; age–GDRS = 0.619*), while its correlation with the outcome anxiety is slightly negative (age–anxiety = -0.059*), whereas GDRS correlates positively with anxiety (GDRS–anxiety = 0.047*). The same applies to depressive symptoms. We summarized the results if Table S5 in the last sentence of Results and included a one-sentence textbook-style note defining suppression: “A suppression pattern arises when the exposure and a covariate are correlated but relate to the outcome in opposite directions, so that adjusting for the covariate increases the absolute exposure–outcome association.” (manuscript with tracked changes lines 213-215)

5) Distributional reporting of the exposure.

You report the geometric mean of the predicted 5-year risk (1.15%). For readers, the median (IQR) and a simple

histogram of predicted risk would be more interpretable; consider adding both. Also, clearly state the scale of the logtransform used in sensitivity analyses (natural log) and report the effect per 1 SD of log-risk for comparability [L185-L192][L249-L257].

**Response:**

We now also report the median (1.21%) and IQR (0.27, 4.81%) of the predicted 5-year risk in the Results (manuscript with tracked changes line 222) of the predicted 5-year risk. To keep the manuscript concise, we did not add a figure; a simple histogram is provided here for the reviewer and can be included as Supplemental Figure in the Supplement if the reviewer and editor prefers.

Regarding the scaling of the sensitivity analyses, we clarify in lines 211-216 (manuscript with tracked changes) that we used the natural logarithm (ln) applied to the predicted 5-year risk on the probability scale (0–1). For comparability, we additionally estimated associations per 1 SD increase in ln-risk by using a z-standardized ln-risk predictor. Results were very similar to those from the per-unit ln-risk specification (S3 Table). To avoid redundancy, we have not added a separate table to the manuscript or supplement.

|  | T2D risk score (logarithmically transformed) per 1 SD |
| --- | --- |
|  | PR^1^ (95% CI) |
| **Very good/good self-rated health (SRH)** |  |
| Model 1 | 0.81 (0.79-0.84) |
| Model 2 | 0.81 (0.79-0.83) |
| Model 3 | 0.79 (0.75-0.83) |
| Model 4 | 0.82 (0.78-0.86) |
| **Excellent/very good self-rated mental health (SRMH)** |  |
| Model 1 | 0.81 (0.77-0.85) |
| Model 2 | 0.79 (0.75-0.84) |
| Model 3 | 0.69 (0.63-0.76) |
| Model 4 | 0.74 (0.67-0.81) |
| **Depressive symptoms** |  |
| Model 1 | 1.15 (1.01-1.30) |
| Model 2 | 1.16 (1.02-1.32) |
| Model 3 | 1.69 (1.43-1.99) |
| Model 4 | 1.44 (1.20-1.73) |
| **Anxiety symptoms** |  |
| Model 1 | 1.15 (0.99-1.34) |
| Model 2 | 1.19 (1.02-1.38) |
| Model 3 | 1.90 (1.57-2.29) |
| Model 4 | 1.66 (1.36-2.03) |

6) Imputed waist circumference inside GDRS.

Waist was estimated from height/weight/age/sex using a prior equation; that’s acceptable, but it can shift risk

categorization. Please (a) state the predictive performance (R²/RMSE) of the equation you used, (b) do a sensitivity analysis replacing GDRS with a version that omits waist (or uses BMI) to show robustness, and (c) discuss potential misclassification bias [L121-L127. The GDRS validation and simplified versions are good anchors to cite.

**Response:** We thank the reviewer for this careful point.

(a) The predictive performance of the used equation was: men: R²=0.86, RMSE=4.86; women: R²=0.82, RMSE=6.05. We added just the R² in the manuscript to avoid overloading the manuscript (manuscript with tracked changes line 127).

(b) In our study, the exposure is the validated German Diabetes Risk Score (GDRS) instrument, which was originally derived and updated using waist circumference (WC), not BMI, as the anthropometric predictor of obesity, together with height and other non-invasive factors. The choice of WC in the GDRS reflects evidence that central adiposity is more strongly predictive of incident type 2 diabetes than BMI, and WC was a key component in the original derivation and updated version, which are cited in the Methods. Because the GDRS has not been validated with BMI in place of WC, we refrain from substituting BMI for WC within the score.

(c) We added a sentence in the discussion regarding the potential bias (manuscript with tracked changes lines 439-442).

7) Statistical reporting format.

Adopt the journal’s preferred style: report PR (95% CI) with consistent decimals and avoid “95%-CI” and “PR1”

notations. PLOS’s stats guidance asks for consistent reporting of regression results and measures of variance. See also PLOS ONE’s submission guidelines (statistical reporting section).

**Response:** Thank you for the guidance. We have harmonized statistical reporting throughout: PRs are now presented with two decimal places, confidence intervals are formatted as “95% CI” (not “95%-CI”), and footnotes use superscript letters (e.g., ᵃ) instead of “PR¹”. These changes have been applied consistently across the text, tables, and figures.

**Presentation (tables/“graphs”)**

Figures: I don’t see any figures/plots in the main text; the manuscript currently presents results via Tables 1–3.

Consider adding a forest plot of adjusted PRs (Model 4) for each outcome with risk categories on the y-axis; this would visually summarize your key findings and is a common, reader-friendly presentation in PLOS ONE.

**Response:** Thank you for this suggestion. We added a forest plot for Model 4 in the results.

Tables (specific fixes):

• Table 1—category label inconsistency. The text defines SRH categories as “very good/good” vs “fair/bad/very bad,” but Table 1 uses “Average/poor/very poor.” Please align wording (use “Fair/Bad/Very bad” or “Fair/Poor/Very poor” consistently) [L31-L37][L131-L137][L135-L141][L135-L141].

• Table 1—typo in confidence interval. Region “South” shows 29.7 (27-7–31.8); should be 29.7 (27.7–31.8)[L155-

L160].

• Table 2—formatting. Use 95% CI not “95%-CI”;

ensure a uniform number of decimals across percentages and CIs, and consider reporting exact p to 3 decimals except where p<0.001 [L204-L207].

• Table 3—footnote style. The column header currently shows “PR1 (95%-CI)” while footnote 1 explains the models.

Change “PR1” in the header to “PR” and keep the superscript/footnote on the table title or at the end (to avoid reading it as a variable name) [L241-L248][L245-L248].

• Model Ns. Provide the analytic N for each model row block (Model 1–4) since excluding covariate missings changes sample size [L243-L248].

**Line-by-line language & style edits (with exact lines)**

Note: PLOS doesn’t enforce US vs UK spelling, but they want consistent English throughout. You currently mix

“behavioural/favourable/organisation/generalised/anonymised” with American variants. I suggest standardizing to one dialect across the whole paper (examples below). PLOS also prefers “95% CI,” sentence case titles, and clean abbreviation definitions.

Title & front matter

• Short title capitalization looks fine. Ensure sentence case for full title (PLOS guideline) [L36-L37].

Keywords (capitalization consistency):

• “anxiety symptoms; Depressive Symptoms; German Diabetes Risk Score; Population-based survey; Subjective health” → make consistent lowercase: “Anxiety symptoms; depressive symptoms; German Diabetes Risk Score; population-based survey; subjective health.” [L41-L43].

Abstract

• “telephone-interview survey” → telephone interview survey [L35-L36].

• Risk categories: replace “>=10%” with ≥10% (Unicode symbol) for consistency with journal style; similarly for other inequality signs [L36-L39].

• “Poisson regression was used … adjusting for …” → “… and models were adjusted for …” (active, parallel wording) [L39-L41].

• “95% confidence interval, CI” → “95% confidence interval (CI)” [L42-L44].

Introduction

• “etiologically heterogenous chronic disorder” → heterogeneous [L46-L51].

• Consistency: Decide on behavioral vs behavioural and use one form; e.g., “behavioral-related” rather than

“behavioural-related” (several instances) [L77-L84].

• Minor: “e. g.” → e.g. (no spaces) [L65-L72].

Methods—Design & participants

• “German speaking resident adult population” → German-speaking [L87-L93].

• “computer-assisted telephone interview (CATI)” already defined—good.

• Ethics tense/consistency: “All participants … must give their verbal consent …” → “… gave their verbal consent …” (past tense) [L101-L107].

• “anonymised transfer” → anonymized (if choosing US spelling) [L103-L107].

• GDPR citation format: “Art. 7 No. 1” → consider “Article 7(1)” for standard legal style [L107-L110].

Methods—Exposure & outcomes

• SRH operationalization: consistent with WHO/MEHM; keep wording aligned with the categories you actually use in tables (see Table 1 point) [L129-L137].

• SRMH: “combined to” → combined into; and your second category title uses “good/fair/poor” while the original response option is “bad”—use “good/fair/bad” or stick to “good/fair/poor” but then reflect that in the prompt text; be consistent across text and tables [L31-L37].

• PHQ-2 / GAD-2 cut-offs: prefer “cutoff” (noun) or “cut-off”; you currently use “cut off” (two words) in several places [L53-L55][L61-L63].

• “Generalised Anxiety Disorder-2 (GAD-2)” → Generalized if adopting US spelling; the instrument name is commonly styled “Generalized Anxiety Disorder-2–item (GAD-2)” in US journals [L57-L61].

Methods—Statistics

• “Rao-Scott-Chi-Square test” → Rao–Scott chi-square test (en dash; lower-case chi-square) [L166-L170].

• “Prevalence Ratios (PR)” → prevalence ratios (PRs); keep pluralization consistent [L171-L179].

• “We utilise Poisson regression …” → utilize or simply use; avoid mixed dialects [L171-L179].

• Spell out design elements of svy (weights/PSU/strata, variance estimator) as noted above.

Results

• Minor discrepancy: text says “A total of 71% self-rated …” while Table 1 shows 72.0%. Harmonize (rounding) [L189-

L192][L173-L180].

• Reporting style: keep a uniform decimal precision across proportions and CIs in text (e.g., one decimal for

percentages; two for CIs), and always as % (95% CI: a–b) [L197-L205].

Tables

• Table 1: fix CI typo 29.7 (27.7–31.8) for South region [10:L155-L160].

• Table 2 & 3: Use 95% CI (no hyphen) and avoid “PR1” in headers; place the footnote marker after the table title instead [L204-L207][L241-L248].

**Response:**

Thank you for the table/style suggestions. We have implemented all requested changes in the manuscript. Regarding decimals, we now report PRs (and 95% CI) with two decimals for comparability, while prevalence estimates are presented with one decimal to avoid spurious precision given their magnitude.

Discussion

• Great that you note suppression and bidirectionality. Consider adding one citation on modified Poisson/log-binomial vs logistic for PRs to help readers less familiar with PR estimation in cross-sectional data.

**Response:** Thank you for the suggestion. We now cite standard references on PR estimation in cross-sectional data in the Methods section, specifically modified Poisson with robust variance versus logistic regression (Coutinho et al., 2008), to aid readers less familiar with these methods.

One-page list of quick textual fixes (by line)

• Heterogenous → heterogeneous [11:L46-L51]

• telephone-interview survey → telephone interview survey [16:L35-L36]

• 95%-CI → 95% CI (everywhere)[12:L204-L207]

• PR1 (95%-CI) → PR (95% CI) (Table 3 header)[14:L241-L248]

• >=10% → ≥10% (risk thresholds)[16:L36-L39]

• must give … consent → gave … consent (past tense)[2:L101-L107]

• anonymised → anonymized (or make all spellings UK; be consistent)[2:L103-L107]

• e. g. → e.g.[13:L65-L72]

• Generalised → Generalized (if US spelling)[1:L57-L61]

• cut off → cutoff / cut-off (noun)[1:L53-L55][1:L61-L63]

• Rao-Scott-Chi-Square → Rao–Scott chi-square[9:L166-L170]

• behavioural-related → behavioral-related (or standardize to UK throughout)[13:L77-L84]

• SRMH categories: ensure text and tables match (avoid “Average/poor/very poor” if the described options are “fair/

bad/very bad”)[1:L31-L37][10:L131-L137]

• Table 1 South CI typo: 29.7 (27.7–31.8)[10:L155-L160]

• 71% vs 72% SRH prevalence: harmonize rounding[9:L189-L192][10:L173-L180]

**Response:** Thank you for the concise suggestions. We have implemented all proposed textual edits and revised the manuscript accordingly; where minor alternatives were possible, we adopted the phrasing consistent with PLOS ONE style and terminology.

**Bottom-line assessment**

• Strengths: Timely national surveillance topic; appropriate use of a validated risk score (GDRS) in a large, weighted

sample; consistent inverse associations for SRH/SRMH and positive associations for depressive/anxiety symptoms;

thoughtful note on suppression by age.

• Main revisions needed: Fully specify regression variance/design, tighten reporting to PLOS style, fix a few table/text inconsistencies/typos, and add small sensitivity analyses (especially around the imputed waist component of GDRS and exposure scaling). A compact forest plot would also upgrade readability.
